# Monocyte urokinase-type plasminogen activator up-regulation reduces thrombus size in a model of venous thrombosis

**DOI:** 10.1016/j.jvs.2009.06.047

**Published:** 2009-11

**Authors:** Julia Humphries, James A. Gossage, Bijan Modarai, Kevin G. Burnand, Thomas H. Sisson, Colin Murdoch, Alberto Smith

**Affiliations:** a King's College London British Heart Foundation Centre, Academic Department of Surgery, Cardiovascular Division, National Institute for Health Research Biomedical Research Centre at Guy's and St Thomas' National Health Service Foundation Trust, London, United Kingdom; b Department of Internal Medicine, University of Michigan Medical School, Ann Arbor, Mich

## Abstract

**Background:**

Our previous studies showed that the direct injection of an adenovirus construct expressing urokinase-type plasminogen activator (uPA) into experimental venous thrombi significantly reduces thrombus weight. The systemic use of adenovirus vectors is limited by inherent hepatic tropism and inflammatory response. As macrophages are recruited into venous thrombi, it is reasonable to speculate that these cells could be used to target the adenovirus uPA (ad-uPA) gene construct to the thrombus. The aims of this study were to determine whether macrophages transduced with ad-uPA have increased fibrinolytic activity and whether systemic injection of transduced cells could be used to target uPA expression to the thrombus and reduce its size.

**Methods:**

The effect of up-regulating uPA was examined in an immortalized macrophage cell line (MM6) and macrophages differentiated from human blood monocyte-derived macrophages (HBMMs). Cells were infected with ad-uPA or blank control virus (ad-blank). Fibrinolytic mediator expression, cell viability, and cytokine expression were measured by activity assays and enzyme-linked immunosorbent assays. Monocyte migration was measured using a modified Boyden chamber assay. A model of venous thrombosis was developed and characterized in mice with severe combined immunodeficiency (SCID). This model was used to study whether systemically administered macrophages over-expressing uPA reduced thrombus size. Uptake of HBMMs into the thrombus induced in these mice was confirmed by a combination of PKH2-labeled cell tracking and colocalization with human leukocyte antigen (HLA) by immunohistology.

**Results:**

Compared with ad-blank, treated HBMMs transduction with ad-uPA increased uPA production by >1000-fold (*P* = .003), uPA activity by 150-fold (*P* = .0001), and soluble uPA receptor (uPAR) by almost twofold (*P* = .043). Expression of plasminogen activator inhibitor (PAI-1) and PAI-2 was decreased by about twofold (*P* = .011) and threefold (*P* = .005), respectively. Up-regulation of uPA had no effect on cell viability or inflammatory cytokine production compared with ad-blank or untreated cells. Ad-uPA transduction increased the migration rate of HBMMs (about 20%, *P* = .03) and MM6 cells (>twofold, *P* = .005) compared with ad-blank treated controls. Human macrophage recruitment into the mouse thrombus was confirmed by the colocalization of HLA with the PKH2-marked cells. Systemic injection of uPA-up-regulated HBMMs reduced thrombus weight by approximately 20% compared with ad-blank (*P* = .038) or sham-treated controls (*P* = .0028).

**Conclusion:**

Transduction of HBBM with ad-uPA increases their fibrinolytic activity. Systemic administration of uPA up-regulated HBBMs reduced thrombus size in an experimental model of venous thrombosis. Alternative methods of delivering fibrinolytic agents are worth exploring.

## Clinical Relevance

The use of thrombolysis in the treatment of acute iliofemoral deep vein thrombosis is not suitable for all patients. Our previous studies have shown that direct injection of an adenovirus construct expressing urokinase plasminogen activator (uPA) into experimental venous thrombi significantly reduced thrombus weight. The systemic use of adenovirus vectors is, however, limited by both their inherent hepatic tropism, which precludes targeted delivery to disease sites, and by the associated host inflammatory response. As macrophages are recruited into venous thrombi, these cells could be used to target uPA gene constructs to the thrombus after systemic administration.

The changes that lead to post-thrombotic syndrome are caused by a combination of valvular damage and persistent venous outflow restriction. Rapid recanalization of veins preserves valve integrity and is associated with a lower incidence of reflux.^1^ Patients with shorter recanalization times have an improved clinical outcome.^2^ It is therefore reasonable to expect that enhancing thrombus resolution may reduce the incidence of post-thrombotic complications.

Venous thrombosis is usually treated by anticoagulation, which reduces the risk of pulmonary embolism, prevents thrombus propagation, and allows slow natural resolution.^3^ Anticoagulants do not, however, promote natural resolution and can cause bleeding complications. Thrombolytic agents and thrombectomy remove thrombus rapidly in some patients who are treated early but their use is limited.^4^ Novel strategies that replace or augment anticoagulation and hasten thrombus resolution without the risk of hemorrhage are therefore attractive. Natural thrombus resolution is regulated by an inflammatory response involving the recruitment of neutrophils and monocytes, followed by endothelial cells and myofibroblasts, leading to thrombus organization, contraction, and recanalization of the vein lumen.^5^

There is a progressive increase in plasminogen activator (PA) activity within the thrombus as organization proceeds that appears to be associated with the influx of monocytes^6^ that are recruited into the thrombus in a temporal pattern.^7^ The fibrinolytic system is an important mediator of cell migration and tissue remodelling.^8^ Net fibrinolytic activity is the product of the relative abundance of PAs and their inhibitors, PAI-1 and PAI-2.^9^

Deletion of the gene encoding the urokinase-type plasminogen activator (uPA) prevents thrombus resolution, and this is associated with a marked reduction in monocyte ingress into the thrombus.^10^ These findings have led to the hypothesis that monocyte recruitment, regulated through the expression of uPA, is an essential mechanism in thrombus resolution.

Direct injection of an adenovirus construct expressing uPA (ad-uPA) into experimental venous thrombi significantly reduces thrombus weight.^11^ The systemic use of adenovirus vectors, however, is limited by their inherent hepatic tropism, which precludes targeted delivery to alternative organs or disease sites, and by the host inflammatory response.^12^ As monocytes are recruited into venous thrombi, it would be reasonable to speculate that these cells could be used to target the ad-uPA gene construct to the thrombus after systemic administration. The aims of this study were therefore to determine whether monocytes transduced with ad-uPA have increased fibrinolytic activity and whether systemic injection of transduced cells could be used to target uPA expression to the thrombus and reduce its size.

## Materials and methods

Permission to conduct these studies was obtained from St Thomas' Hospital Ethics Committee. Serotype-5 adenoviral vectors encoding human uPA (ad-uPA)^13^ and an empty cassette (ad-blank) were used. Adenovirus was amplified in human embryonic kidney cells (HEK-293), and purified and titered using the AdenoX system (Becton Dickinson, Franklin Lakes, NJ).

### Transduction of human blood monocyte-derived macrophages

Pure (>90%) monocyte preparations were isolated from blood using Optiprep (Axis-Shield, Cambridgeshire, UK).^14^ Isolated cells were resuspended in Roswell Park Memorial Institute media (Sigma Aldrich, Dorset, UK) supplemented with 10% fetal calf serum (Life Technologies, Paisley, UK) and 2 mM glutamine (Sigma). Monocytes/macrophages are resistant to adenovirus infection.^15^ Cells were therefore cultured for 72 hours with 100-ng/mL monocyte colony-stimulating factor (MCSF; R&D Systems, Abingdon, UK) to up-regulate integrins, which facilitate adenoviral infection. Cells were treated with the adenovirus or culture media alone (baseline control) at a multiplicity of infection (MOI) of up to 2500 and harvested after 4 days. Cell viability was assessed by measuring lactate dehydrogenase (LDH; Promega, Southampton, UK) and caspase-3 activity (Sigma) in conditioned media.

### Confirmation of human blood monocyte-derived macrophages transduction

Urokinase upregulation was confirmed before use in all studies as follows:

• *uPA fibrin plate analysis*: A fibrin gel was formed using fibrinogen (0.4%, Sigma), agarose (0.15%, Sigma), and lys-plasminogen (25 μg/mL) in phosphate-buffered saline (pH 7.4; Sigma). Fibrin cross-linkage was induced by the addition of thrombin (5 IU/mL). Conditioned media and active uPA standards were added and incubated for 12 hours at 37°C.
• *uPA S2444 substrate activity assay:* Urokinase (uPA) activity was measured using a colorimetric assay.^16^

### Fibrinolytic activity and inflammatory mediator expression after adenovirus transduction

Monocytes were isolated from 12 donors and transfected with 1000 MOI of ad-uPA, ad-blank, or carrier alone (baseline control). Levels of uPA activity and uPA antigen were measured in human blood monocyte-derived macrophages (HBMM)-conditioned media using a colorimetric assay^16^ and enzyme-linked immunosorbent assay (ELISA),^17^ respectively. The levels of PAI-1,^18^ PAI-2,^19^ soluble uPA receptor (uPAR, R&D Systems), tissue plasminogen activator (tPA; gift, Prof. P. J. Declerck, Leuven, Belgium), and monocyte chemotactic protein-1 (MCP-1, R&D Systems) were measured by ELISA. Tumour necrosis factor-α (TNF-α), regulated upon activation normal T-cell expressed and secreted (RANTES), interferon-γ (IFN-γ), interleukin-1α (IL-1α), IL-1β, IL-4, IL-6, IL-8, IL-10, and granulocyte macrophage-colony stimulating factor (GM-CSF) were measured using the Beadmaster system (Upstate, USA, Millipore, Billerica, Mass).

### Ad-uPA transduction and monocyte migration in vitro

A macrophage cell line, MM6 (gift, Professor Ziegler-Heitbrock, Munich, Germany), and HBMMs were transfected with 1000 MOI of ad-uPA or ad-blank as described. Cell migration was assessed using a modified Boyden chamber.^20^ Cells were fluorescently labeled with Calcein AM (Invitrogen, Paisley, UK).

Filter inserts (3-μm pore size, Fluoroblok; BD Biosciences, Becton Dickinson, Franklin Lakes, NJ) were coated in fibrin gel to a depth of 1 mm. Macrophages (5 × 10^5^ cells) were added to the upper chamber, and MCP-1 (300 ng/mL, R&D Systems) or carrier was added to the lower chamber. Fluorescence was measured over 150 minutes (VictorLight; Perkin-Elmer, Salem, Mass).

Experiments were performed in quintuplicate with MM6 cells and in triplicate with HBMMs. The data were expressed as the percentage of maximal fluorescence obtained from the total number of labeled cells used.

### Severe combined immunodeficiency mouse model of venous thrombosis

UK Home Office approval was obtained for all animal procedures. Thrombosis was induced in the inferior vena cava of severe combined immunodeficiency (SCID) mice using reduced flow and endothelial disturbance.^10,11^ Thrombi were harvested at 1, 7, 14 and 21 days after induction. Paraffin sections (5 μm) were taken at 300-μm intervals throughout the whole length of the thrombus. The area of thrombus over a defined length gives an approximation of thrombus volume.^10^ Thrombus and recanalization areas were measured in sections stained with hematoxylin and eosin using image analysis.^21^

Neutrophil ingress (identified by nuclear morphology) was assessed in hematoxylin and eosin sections taken from 1-day-old thrombi. Lymphocytes were stained with anti-CD3, a T-cell marker, and anti-CD79a, a B-cell marker (both Abcam, Cambridge, UK).

Monocyte ingress occurs at a later stage.^7^ Sections from 7- and 14-day thrombi were therefore stained with the mouse monocyte marker MAC3 (BD Pharminogen, Becton Dickinson, Franklin Lakes, NJ). Primary antibody binding was located using biotinylated secondary antibodies and the ABC-peroxidase complex (Vector Laboratories, Peterborough, UK). Matched isotype or polyclonal immunoglobulin from the appropriate species acted as controls. Twenty sections of each thrombus were evaluated for each stain or control. Staining was quantified by image analysis and calculated as a mean percentage of the total area.

### Tracking of HBMMs injected into SCID mice

HBMMs were isolated and labeled with PKH2 fluorescent cell marker as previously described.^22^ Labeled cells (6.5 × 10^5^) were injected into the tail vein of the SCID mouse 2 hours before thrombus formation. The thrombi were removed after 7 days. PKH2 green fluorescence was colocalized with the binding of a red fluorescently labeled anti-human leukocyte antigen (HLA) antibody (Serotec, Kidlington, UK). Primary antibody was detected with an antimouse Alexa 568 conjugated secondary antibody (Cambridge Biosciences, Cambridge, UK). An isotype-matched antibody was used as a negative control.

### The effect of uPA up-regulation in HBMMs on thrombus resolution

HBMMs (1 × 10^6^ cells) were transfected with 2500 MOI of ad-uPA or ad-blank and injected 2 hours before thrombus formation into the tail vein of two cohorts of eight mice. A third cohort of eight animals was injected with carrier containing no cells. The thrombi were harvested 7 days after induction, in a blinded manner. At 7 days, the model thrombus in untreated controls is still occlusive but beginning to organize; therefore, any effect of treatment would be expected to be maximal at this point.^23^

### Statistical analysis

Values were expressed as mean ± standard error (SEM). Two-way analysis of variance (ANOVA) was used to compare the temporal changes in thrombus resolution in the SCID model and wild-type controls. Paired and unpaired *t* tests were used for comparisons of parametric data and Wilcoxon or Mann Whitney *U* tests were applied to nonparametric data.

## Results

### HBMM fibrinolytic mediator production and cytokine response after ad-uPA transduction

Ad-uPA transduction increased HBMM fibrinolytic activity by >150-fold (*P* = .0001) compared with ad-blank–treated controls (Table I). Production of uPA antigen was increased from the lower limit of detection of the uPA ELISA by >1000-fold (*P* = .003, Table I). No tPA antigen or activity could be measured in treated HBMMs. Up-regulation of uPA resulted in a 1.6-fold increase in HBMM uPAR production (*P* = .043); almost 1.6-fold decrease in PAI-1 production (*P* = .011); and a greater than twofold decrease in PAI-2 production (*P* = .005) compared with ad-blank controls. No significant difference between ad-blank and baseline (carrier alone) control groups was shown for any of the fibrinolytic mediators measured (Table I).

Five of 10 cytokines analyzed were detectable (IL-8, IL-6, IFN-γ, RANTES, GM-CSF), and the expression of these cytokines was not significantly different between baseline and cells treated with ad-uPA or ad-blank (Table II). Levels of IL-1α, IL- 1β, TNF-α, IL-4, and IL-10 could not be measured. LDH release and caspase-3 activity could not be measured in untreated (baseline) cells or cells treated with ad-uPA.

### Effect of monocyte uPA up-regulation on in vitro MM6 and HBMM migration

**Fig 1:**
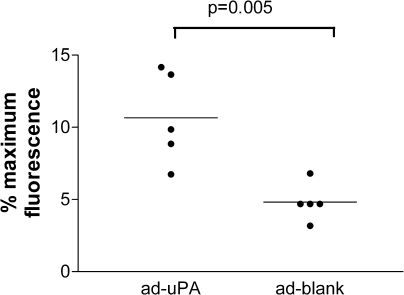
Migration of MM6 cells treated with either adenovirus *(ad)* expressing urokinase-type plasminogen activator *(ad-uPA)* or ad-blank across a fibrin matrix (n = 5). Each experiment was performed in quintuplicate (*bars* represent means). Transfection with ad-uPA resulted in a more rapid migration of MM6 cells across fibrin-coated filters (11% [standard error of the mean, 1.4%]) compared with ad-blank–treated cells (5% [standard error of the mean, 0.6%], *P* = .005).

**Fig 2:**
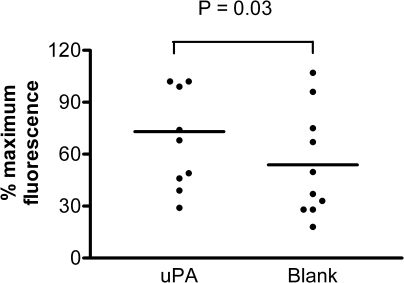
Migration of primary monocytes treated with either adenovirus *(ad)* expressing urokinase-type plasminogen activator *(ad-uPA)* or ad-blank across a fibrin matrix (n = 10). Each experiment was performed in triplicate (Bars represent means). Treatment with ad-uPA also resulted in the accelerated movement of cells across fibrin-coated filters (70% [standard error of the mean, 8.9%]) compared with ad-blank–treated cells (54% [standard error of the mean, 9.8%], *P* = .03).

Transduction with ad-uPA resulted in a more rapid migration of MM6 cells across fibrin-coated filters (11% ± 1.4%) compared with ad-blank–treated cells (5% ± 0.6%, *P* = 0.005, Fig 1). Treatment with ad-uPA also resulted in the accelerated movement of HBMMs cells across fibrin-coated filters (70% ± 8.9%) compared with ad-blank–treated cells (54% ± 9.8%, *P* = .03, Fig 2).

### Characterization of thrombus resolution in SCID mice

Laminar thrombi were successfully formed in all mice with no complications. There was no difference in the thrombus size between the SCID mice and their wild-type controls at 24 hours (11.4 ± 0.8 vs 12.3 ± 1.6mm^2^), 1 week (10.2 ± 1.7 vs 12.9 ± 1.3 mm^2^), 2 weeks (2.5 ±0.6 vs 5.5 ± 2.3 mm^2^), or 3 weeks (1.7 ± 0.3 vs 1.6 ± 0.7 mm^2^; two-way ANOVA, *P* > .1, n = 8/group); ad hoc post-testing also showed no difference in thrombus size at any time interval. The number and distribution of neutrophils in the infiltrate that occurred during thrombogenesis was similar in the SCID mice (8.4% ± 0.8% of thrombus area compared with 7.1% ± 1% in wild type controls, *P* > .1, n = 8/group). There was also no difference in the area of thrombus recanalization or the number of infiltrating monocytes found in the two groups during resolution (ANOVA, *P* > 0.1, n = 8/group).

No difference was found in the speed and size of vein lumen recanalization between the SCID and their wild-type controls at 1 week (3.5 ± 0.5 vs 5.4 ± 1.0 mm^2^), 2 weeks (11.4 ± 1.3 vs 11.3 ± 1.4 mm^2^), or 3 weeks (22.9 ± 1.8 vs 21.9 ± 1.7 mm^2^; two-way ANOVA, *P* > 0.1).

### Tracking of injected HBMMs

**Fig 3:**
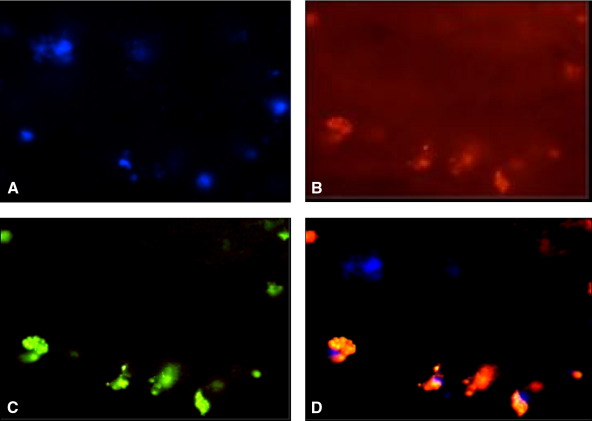
Recruitment of systemically injected monocytes in the thrombus. **A**, Nuclear stain, *blue fluorescence*. **B**, Immunohistochemical staining human leukocyte antigen, *red fluorescence*. **C**, PKH2-labeled cells, *green fluorescence*. **D**, Composite staining of PKH-labeled human monocytes with antihuman leukocyte antigen (original magnification ×400).

More than 90% of HBMMs were labeled with PKH2. Fluorescently labeled (PKH2) HBMMs were present in the thrombus at 7 days after intravenous injection. There was negligible fluorescence in the vein wall at this time. The PKH2-labeled cells costained positively for HLA (Fig 3).

### Effect of monocyte uPA up-regulation on thrombus resolution

**Fig 4:**
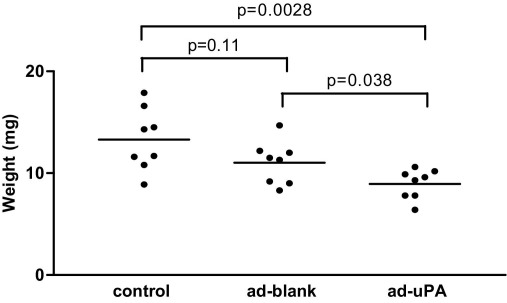
Intravenous injection of adenovirus *(ad)* urokinase-type plasminogen activator (*uPA*) up-regulated human blood monocyte-derived macrophages (HBMMs) reduced thrombus weight at 7 days by approximately 20% (9.0 mg [standard error of the mean] 0.52 mg) compared with either ad-blank–treated HBMMs (11.0 mg [standard error of the mean, 0.74 mg] *P* = .038) or control animals injected with carrier alone (13.3 mg [standard error of the mean, 1.08], *P* = .0028). N = 8 in each group. The *bars* represent means.

No evidence of any immune rejection was noted, and animals appeared to be healthy for the duration of the study. No pathologic bleeding or premature deaths occurred. Systemic injection of uPA-upregulated HBMMs reduced thrombus weight at 7 days by about 20% (9.0 ± 0.52 mg) compared with ad-blank–treated HBMMs (11.0 ± 0.74 mg, *P* = .038) and control animals injected with carrier alone (13.3 ± 1.08 mg, *P* = .0028, Fig 4).

## Discussion

To our knowledge, this is the first report to show that adenovirus-mediated up-regulation of uPA in monocytic cells reduces the weight of experimentally induced venous thrombi. Transduction of HBMMs with ad-uPA resulted in >150-fold increase in uPA-mediated fibrinolytic activity compared with ad-blank–transfected cells, without affecting their viability or inflammatory responses in vitro. The increased uPA activity was observed 4 days after transduction in vitro. We have previously shown that direct injection of the construct used in this study results in increased uPA activity in the thrombus for at least 7 days,^11^ while others have reported that this construct expresses active uPA for at least 14 days in vivo.^13^

The increased fibrinolytic activity of ad-uPA up-regulated HBMMs was also associated with a modest but significant rise in the expression of uPAR and a reduction in PAI-1 and PAI-2 protein levels. The higher levels of soluble uPAR produced by the cells treated with ad-uPA may have reflected the cleavage of uPAR at the cell surface by the increased amounts of uPA expressed. Urokinase mediates the cleavage of uPAR as part of a negative feedback mechanism to regulate activation of plasminogen on the surface of monocytic cells.^24^ The reduced levels of PAI-1 and PAI-2 cannot be explained by the inability of the ELISAs to discriminate between PAI:uPA complexes, because there was no difference in the ELISA signal generated after the addition of excess PAI to a standard amount of uPA compared with uPA alone (data not shown). The reduced expression of both PAI-1 and PAI-2 after transduction with the ad-uPA construct may have been the result of their internalization by endocytosis after inhibition of uPA:uPAR complexes by either of these PAIs.^25^ Levels of the uPAR would be less affected by this process as it is subsequently recycled to the cell surface.^25^

Treatment of isolated HBMMs with ad-uPA did not appear to induce an inflammatory response. This finding is similar to that obtained after ad-tPA transduction of human mononuclear cell isolates^26^ but contrasts with studies on the effect of general adenoviral infection on mononuclear cell responses.^27^ It is difficult, however, to compare these studies because there are a number of differences in the methods used, including different isolation procedures and cell pre-treatments that may affect the expression of inflammatory mediators by macrophages. Some of the cytokines assessed in this study could not be measured because they fell below the sensitivity of their respective assays. It is possible that there were minor differences between treatments that we could not detect. No difference was found between ad-uPA, ad-blank, or uninfected cells in the levels of inflammatory cytokines that were measurable.

The model of venous thrombosis in the SCID mouse was based on our previous mouse models.^10,11^ Thrombus formation was highly reproducible, whereas the pattern of resolution was similar to that seen in wild-type controls, suggesting that lymphocytes do not participate in thrombus organization. The absence of lymphocytes did not appear to affect the number and distribution of other leukocytes found within the resolving thrombi. Neutrophils, which are the most common type of inflammatory cells found in the thrombus at an early stage after its formation,^28^ were present in similar numbers in the SCID mice and their wild-type controls. Neutrophils have both fibrinolytic^29^ and antifibrinolytic activity^30^ that appears to regulate thrombus size.^28^

A similar temporal and spatial distribution of monocyte recruitment and revascularization was also seen in the thrombi produced in SCID mice compared with their wild-type controls. The model allowed us to use human monocytes, which are more easily isolated, in larger numbers and with greater purity than rodent monocytes. This negated the use of large numbers of donor animals.

Because the binding of uPA to its receptor is species specific, it was necessary to transduce the HBMMs with the human uPA gene.^13^ A weakness in this approach is that although human uPA can activate mouse plasminogen, it does this with less efficiency than human plasminogen (data not shown). The use of murine cells up-regulated with murine uPA was, however, precluded because the isolation of pure populations of these cells in sufficient numbers was not possible. It is conceivable, however, that a larger effect on thrombus size might have been achieved if murine macrophages upregulated for murine uPA had been used.

We have previously demonstrated that direct injection of macrophages into the thrombus significantly reduces thrombus size.^31^ These cells may stimulate resolution by expressing a variety of enzymes, growth, and chemotactic factors that stimulate organization and recanalization.^7,32^ Our previous work has shown that systemically injected rodent mononuclear cells are recruited into resolving thrombus.^7^ This study provides evidence that systemically injected human monocytes (HBMMs) are also recruited into the thrombus and that up-regulating uPA expression in these cells results in enhanced resolution. There are several plausible explanations for this effect:

First, uPA accelerated monocyte migration and delivered fibrinolytically active monocytes much earlier to the thrombus. The in vitro studies showed a faster migration rate for both HBMMs and MM6 cells transfected with ad-uPA, confirming the work of others.^33^

Urokinase has a well-documented action on surface proteolysis, cell adhesion, chemotaxis, and migration and these effects have been noted both in vitro and in vivo.^10,33,34^ Mice lacking the uPA gene fail to recruit cells (monocytes in particular) into the thrombus.^10^ Urokinase-dependent conformational change in uPAR mediates cytoskeletal changes, activation of kinases, and directional cell migration.^34^ Urokinase also generates plasmin by cleaving the proenzyme plasminogen. Plasmin is not only fibrinolytic but also activates other proteases that are crucial for cell migration in tissues.^35^

Reliable quantification of the numbers of HBMMs recruited was, however, difficult because of the background fluorescence associated with this label. Attempts at quantifying HBMM numbers using reverse transcription-polymerase chain reaction were not successful. Better methods of tracking injected monocytes using reporter gene technology in combination with imaging methods such as single photon emission computed tomography (SPECT/CT) or micro-PET would allow a sensitive, noninvasive (longitudinal), quantitative tracking of injected ad-uPA-transduced monocyte recruitment during resolution.

Secondly, these cells may have promoted resolution by enhanced fibrinolysis mediated by the extra uPA generated within the thrombus. This is in keeping with our previous work showing that the induction of picogram quantities of uPA activity within the thrombus (generated from direct ad-uPA injection) is sufficient to increase the resolution of the thrombus.^11^

Thirdly, it is also possible that ad-uPA-transduced monocytes accelerated resolution through other mechanisms that could include increasing endogenous monocyte influx by releasing chemotactic cytokines at an early stage in resolution.

The development of methods capable of quantitatively tracking both endogenous and exogenously administered monocytes would help to define how these cells exert their effect on thrombus resolution.

Altered macrophages have been used therapeutically in humans for the treatment of malignant disease^36^ and might also be used as gene delivery vehicles because they have a natural tendency to migrate to the site of disease, such as the thrombus. Using cells that have been altered by ex vivo manipulation may obviate some of the problems of administering virus alone, including rapid loss to the liver and lack of penetration into tissue.^12^ Targeting transduction to circulating monocytes may also be possible by coating the viral particles with sugars that bind to specific receptors on these cells.^37^ This would prevent loss of the adenovirus to the liver after systemic administration.

One study suggested that up-regulation of macrophage uPA might enhance atherogenesis,^38^ but that study was done in animals whose macrophages constitutively over-expressed uPA. The method of uPA up-regulation used in this study is transient (approximately 15 days) and is therefore unlikely to cause long-term problems.

Further studies are required to determine whether:

1 the time of delivery of the fibrinolytically overactive macrophages to the thrombus is important;
2 injected monocytes enhance resolution directly or indirectly by promoting endogenous monocyte recruitment;
3 the effect of concomitantly up-regulating other factors, known to increase uPA activity, such as its substrate plasminogen could enhance their ability to resolve thrombus;
4 the use of more powerful viral promoters that generate higher and more sustained levels of uPA production have greater efficacy; and
5 it is possible to reduce loss of injected cells to other tissue sinks.

## Conclusions

This study provides evidence that up-regulating monocyte uPA enhances thrombus resolution. Our previous studies have shown that thrombus resolution can be enhanced through the direct injection of monocytes^7,31^ or adenovirus constructs over-expressing uPA^11^ or vascular endothelial growth factor.^23^ Increasing the circulating concentrations of monocyte chemotaxins and angiogenic agents may be counterproductive because they could reduce recruitment of monocytes and other cells important in thrombus organization by overwhelming chemotactic gradients. Monocytes could act as vectors that allow more specific targeting of the thrombus. The proteins that are up-regulated and the means by which this is achieved would obviously require refinement.

There is also increasing evidence to suggest that macrophages display phenotypic heterogeneity, with at least two subtypes that are associated with either proinflammatory activity (M1-type macrophages) or remodelling (M2-type) processes.^39^ All monocytes isolated and used in this study were pretreated with MCSF, which is thought to induce an M2 phenotype.^40^ We do not know, however, whether viral transduction or the thrombus milieu had an effect on this.

The development of any treatment that uses exogenous altered cells, promotes their number through bone marrow stimulation, or alters the activity of endogenous circulating cells will rely on a better understanding of how these phenotypes contribute to thrombus resolution.

## Author contributions

Conception and design: JH, JG, MB, KB, AS
Analysis and interpretation: JH, JG, MB, AS
Data collection: JH, JG, MB, CM
Writing the article: JH, JG, AS
Critical revision of the article: JH, JG, TS, KB
Final approval of the article: JH, TS, AS
Statistical analysis: JH, JG, CM, AS
Obtained funding: JH, JG, AS
Overall responsibility: AS

## Figures and Tables

**Table I:** Expression of fibrinolytic mediators by primary human blood-derived monocyte/macrophages transfected with ad-uPA and ad-blank and baseline cells treated with carrier alone

Variables^a^	Ad-uPA	Ad-blank	Baseline	Ad-blank vs ad-uPA, *P*	Ad-blank vs baseline, *P*
uPA antigen, ng/mg sol protein	1122 (297)	0.08 (0)	0.08 (0)	.003	>.90
uPA activity, IU/mL	148 (14)	0.5 (0)	0.5 (0)	.0001	>.90
uPAR, ng/mg sol protein	6684 (2300)	4010 (1243)	4061 (1264)	.043	.68
PAI-1, ng/mg sol protein	17 (5)	27 (6)	23 (7)	.011	.17
PAI-2, ng/mg sol protein	2285 (661)	4913 (1097)	3789 (745)	.005	.07

*Ad*, Adenovirus; *NS*, not significant; *PAI*, plasminogen activator inhibitor-1; *uPA*, urokinase-type plasminogen activator; *uPAR*, urokinase plasminogen activator receptor.

a Data are expressed as mean (standard error of the mean) of 12 healthy individuals.

**Table II:** Expression of cytokines by primary human blood-derived monocytes/macrophages transfected with ad-uPA, ad-blank, and baseline cells treated with carrier alone

Cytokine^a^	Ad-uPA	Ad-blank	Baseline
RANTES, pg/mg sol protein	256 (99)	261 (101)	180 (73)
IL-8, ng/mg sol protein	63 (36)	50 (28)	46 (23)
IFN-γ, pg/mg sol protein	391 (135)	314 (100)	355 (123)
Il-6, pg/mg sol protein	2887 (1886)	2456 (1575)	1889 (1245)
GM-CSF, pg/mg sol protein	792 (333)	739 (283)	579 (225)

*Ad*, Adenovirus; *GM-CSF*, granulocyte macrophage-colony stimulating factor; *IL*, interleukin; *INF*, interferon; *RANTES*, regulated upon activation normal T-cell expressed and secreted; *uPA*, urokinase-type plasminogen activator.

a Data are expressed as mean (standard error of the mean) of 12 healthy individuals.
